# Stillbirth rates and associated risk factors at the Buea and Limbe regional hospitals, Cameroon: a case-control study

**DOI:** 10.1186/s12884-020-2767-8

**Published:** 2020-02-03

**Authors:** Thomas Obinchemti Egbe, Edwin Nkwelle Ewane, Nicholas Tendongfor

**Affiliations:** 1 Department of Obstetrics and Gynaecology, Faculty of Health Sciences, University of Buea, P.O. Box 63, Buea, Cameroon; 2 Department of Obstetrics and Gynaecology, Douala Referral Hospital, Douala, Cameroon; 3 Faculty of Health Sciences, University of Buea, Buea, Cameroon; 4 Department of Public Health and Hygiene, Faculty of Health Sciences, University of Buea, Buea, Cameroon

**Keywords:** Stillbirth rates, Associated risk factors, Case-control study, Buea and Limbe regional hospitals, Cameroon

## Abstract

**Background:**

Stillbirths bring grief to both mother and family but have been underreported in Cameroon. We aimed at determining the stillbirth rates and associated risk factors in the Buea Regional Hospital (BRH) and the Limbe Regional Hospital (LRH), Cameroon.

**Materials and methods:**

This was a hospital-based unmatched case-control study in which files of women with stillbirth (cases) were analysed. A woman with a live birth in the same maternity during the same period served as a control. Data were collected using a pre-tested questionnaire. The stillbirth rate was the number of stillbirths per thousand births. Logistic regression analysis was used to identify independent factors associated with stillbirth.

**Results:**

Stillbirth rates at the BRH and LRH were 33.72 and 36.45 per 1000 live births. The factors that were independently associated with stillbirth were: referral status (AOR 3.95; 95% CI: 1.85–6.58, *p* = 0.000), late booking visit - after 12 weeks (AOR 13.64; 95% CI: 1.49–124.83, *p* = 0.021), preeclampsia (AOR 3.21; 95% CI: 1.23–8.35, *p* = 0.01), placental abruption (AOR 21.46; 95% CI: 2.36–194.77, *p* = 0.006), moderate anaemia (AOR 2.04; 95% CI: 1.09–3.83, *p* = 0.03), labour dystocia (AOR 5.37; 95% CI: 1.77–15.92, *p* = 0.003), low birth weight (AOR 5.27; 95% CI: 1.48–3.53, *p* = 0.000), and preterm delivery (AOR 2.81; 95% CI: 1.48–3.35, *p* = 0.002).

**Conclusion:**

Stillbirth rates are high in both health facilities. Risk factors of stillbirths include referral from another health facility, anaemia, preeclampsia and late booking visit, placental abruption, labour dystocia, preterm birth, and low birth weight. Term, post-term and macrosomia were protective of stillbirth. We recommend electronic data storage in hospitals in Cameroon.

## Introduction

A stillbirth represents a death that occurs before the birth of a baby weighing ≥1000 g, or ≥ 28 completed weeks of gestation, or body length ≥ 35 cm (This definition is used for international comparison: the International Classification of Diseases (ICD) of the World Health Organization uses birth weight ≥ 500 g or ≥ 22 completed weeks of gestation, or body length ≥ 25 cm) [1]. A **“**macerated” stillbirth shows skin and soft tissue changes as discolouration, darkening, redness, peeling and breakdown; suggesting death well before delivery (antepartum) [2]. Fresh stillbirths lack these changes and are presumed to have occurred in less than 12 h (intrapartum) [3]. Stillbirth is still a neglected tragedy. In 2014, the Every NewBorn Action Plan (ENAP) set a target for national Stillbirth Rates (SBRs) of 12 or fewer stillbirths per 1000 births in all countries by 2030 [1, 4]. Stillbirths are associated with psychosocial complications and economic conditions related to funeral costs and loss of earnings due to time off work [3]. Despite the devastating effects, more than 2.6 million stillbirths are still recorded globally every year with very slow progress made to tackle the ‘silent problem’ [4, 5]. The burden of the problem is highest in developing countries. The incidence rates are highest in sub-Saharan Africa [6]. About 98% of all stillbirths occur in Low-Income Countries (LIC) and Middle-Income Countries (MIC). It is estimated that at the current rate of progress to tackle the problem, it will take more than 160 years for a pregnant woman in Africa to have the same chance of her baby being born alive as a woman in a High-Income Country (HIC) today [5].

Several studies have been carried out to estimate the rates of stillbirth in different countries. The rates have been shown to vary widely across the globe: in high-income countries, for example, stillbirth rates in 2006 were estimated at 5 per 1000 births, which are quite low compared to about 32 per 1000 births in South Asia and sub-Saharan Africa [5, 7]. The available data, therefore, suggest higher stillbirth rates in developing countries. In Cameroon, there are few studies on stillbirth, most of which are centred on intrapartum stillbirths [8, 9]. One of these studies reported an incidence of intrapartum foetal death of 9.3 for 1000 live births [9]. There is a dearth of data on the overall stillbirth rate in Cameroon. A stillbirth rate of 25.8%o was reported in Douala in 2017 [10]. There are no studies in semi-urban settings like Buea and Limbe. This study aimed at assessing the rates and risk factors of stillbirth at BRH and LRH.

## Materials and methods

### Study design and setting

This was a 1:3 hospital-based unmatched case-control study conducted from November 2018 to June 2019 at the maternity units of the BRH and LRH. The study included women who gave birth to a stillbirth (cases) at the BRH or LRH from January 2009 to February 2019. Three corresponding live births from 3 days before to 3 days after the index case from the same population served as controls to minimize selection bias. Women who did not give birth in hospitals and those with incomplete files were excluded from the study.

Buea and Limbe Regional Hospitals are the main referral secondary care centres found in the fastest growing towns in the South-West Region, which attract people from all walks of life. Buea is the regional headquarter of the southwest region. It is located at the eastern slopes of Mount Cameroon. It has an estimated population of above 90,088 inhabitants (2005 census) and a surface area of about 870 square Km. Buea health district is divided into seven health areas namely Bova, Buea Town, Bokwango, Tole, Buea Road, Muea and Molyko health areas. The main health facilities in the health area include Integrated Health Centre Bokwango, Muea District Hospital, Seventh Day Adventist Health Centre, St Monica Health Canter, St Veronica Medical Centre, Solidarity Health Foundation, Bebetta Memorial Community Hospital and Mount Mary Hospital. Each of these hospitals conducts an average of five deliveries per week. BRH serves as their main referral hospital. BRH is situated about 20 km from mile 17 Motor Park (the main entrance to Buea). BRH has a maternity unit with a capacity of 13 beds with three beds in the labour room. There are five (5) trained nurses; five (5) midwives and five (5) volunteer midwives. There are three obstetrician/gynaecologists and one general practitioner in the unit. The maternity unit conducts about 1059 deliveries per year with a cesarean section rate of 20.3%.

Limbe is a seaside town located in the southern slopes of Mount Cameroon and is the headquarters of Fako Division in the South West Region of Cameroon. It has a population of about 84,223 inhabitants (2005 census) and a surface area of 674 square km. Limbe health district is divided into eight health areas: Bota, Bojongo, Idenau, Moliwe, Mabeta, Limbe Sea Port, Zone 2, and Batoke health areas. The main health facilities in Limbe are Limbe District Hospital, Presbyterian Health Centre, Limbe Sub-divisional Hospital, Batoke Health Centre, Bonadikombo Integrated Health Centre, Viktoria Hope Foundation Medical Centre, Centre de Sante Inter-Urbain, and Tchenko Clinic. These health facilities conducted an average of 208 deliveries each per year and have as a main referral centre LRH. LRH is located at mile 1, about 13 km from the mile 4 Motor Park. The Regional Hospital in Limbe has a maternity unit with a capacity of twenty-six (26) beds. There are eight (8) midwives, seven (7) nurses and two (2) obstetrician/gynaecologists. The maternity conducts about 1200 deliveries per year.

BRH and LRH serve as teaching hospitals for the Faculty of Health Sciences, University of Buea and provide emergency obstetric and neonatal care.

Both maternity units are equipped with instruments such as foetoscopes, foetal hand-held Doppler, and delivery beds. However, there are no instruments for continuous electronic intrapartum monitoring. Furthermore, the hospitals have well equipped operating theatres, blood banks, laboratories, and imaging units.

### Data collection and analysis

Using the difference in proportions formula, =0.84 for a power of 80%, a significance of 0.05, Za = 1.96, proportion of cases exposed (estimated as 0.26 from a previous study) [11] and a ratio of 1:3, a minimum sample size of 576 (144 cases and 432 controls) was calculated.

A data collection form was developed and used to collect data from the selected files.

Files of cases (stillbirths) and their corresponding controls each (life births in the same period) that met the inclusion criteria were sorted out following consecutive sampling.

The data collection form consisted of three sections: Socio-demographic characteristics; Mother’s reproductive-obstetrical information; and maternal behaviours. To ensure confidentiality, participants’ personal information was coded. The outcome variables were stillbirth (fresh and macerated) and live birth. These were obtained from the delivery registers. The predictor variables were age, referral status, marital status, level of education, occupation, place of residence, parity, gestational age, number of antenatal care visits, gestational age at booking visit, haemoglobin concentration, number of babies, weight of baby, mode of delivery, duration of labour, history of stillbirth, medical illness, infections diagnosed during pregnancy (like malaria and HIV), obstetrical complications, cigarette smoking, and alcohol consumption during pregnancy. These were all obtained from the patients’ files. Mode of delivery, level of education and place of residence were potential confounding variables.

The data were entered into a predesigned template in Epi info 7 and then exported to SPSS version 25 for analysis. Continuous variables such as age, GA, parity, number of antenatal care visits, gestational age at booking visit, and birth weight were categorized. All the missing data were coded but were not included in the final analysis.

The stillbirth rate was described as the number of stillbirths per 1000 births.

The risk factors associated with stillbirth were also described. A logistic regression analysis was used to identify demographic, medical and obstetrical risk factors independently associated with stillbirth. We adhered to STROBE guidelines/methodology.

### Definition of term

A stillbirth is defined as birth with no sign of life, weighing at least 1000 g or at least 28 completed weeks of gestation.

## Results

For 10 years, there were 245 stillbirths out of 7266 deliveries at the BRH and 245 stillbirths out of 6721 deliveries at the LRH. Therefore, stillbirth rates at the BRH and LRH were 33.72 and 36.45 per 1000 births respectively. The average stillbirth rate in both hospitals was 35.03 per 1000 births.

We retrieved a total of 387 files of cases but only 148 (38.24%) met our inclusion criteria. About 67.76% of the files (cases that were either empty or lacked necessary information) were excluded. However, 444 corresponding controls were included in the final analysis. The age of participants ranged from 13 to 43 years with a mean of 27.65 ± 5.30 (cases) and 27.43 ± 5.50 (control). About three (5.6%) of cases had barely attained primary education while 51(94.4%) had attained at least secondary education. Furthermore, 7 (4.3%) of the control group had barely attained primary education and 156 (95.7%) had attained at least secondary education. Barely 15 (10.8%) of cases were employed while 63 (45.3%) were self-employed and 61 (43.9%) were unemployed. Meanwhile, for controls, 80 (19.0%) were employed, 150 (19.0%) were self-employed and 190 (45.2%) were unemployed. The demographic characteristics are presented in Table 1.

**Table 1:** Demographic characteristics

Variable	Categories	N	Percentage
Age group	≤19 years	38	6.4
	≥35 years	61	10.3
	20–34 years	493	83.3
	Total	592	100.0
Marital status	Single	195	32.9
	Married	397	67.1
	Total	592	100.0
Level of education	Primary school	10	4.6
	Secondary school	115	53.0
	Tertiary	92	42.4
	Total	217	100.0
Place of residence	Rural community	122	20.6
	Urban community	470	79.4
	Total	592	100.0
Employment status	No	288	48.6
	Yes	304	51.4
	Total	592	100.0

### Sociodemographic risk factors of stillbirth

After univariate analysis, referral from another health facility increased the likelihood of having a stillbirth 5-fold (OR 5.27; 95%CI: 3.25–8.57, *p* = 0.000) (Table 2).

**Table 2:** Sociodemographic factors associated with stillbirth (bivariate analysis)

Variables	Categories	Case N (%)	Control N (%)	OR (95% CI)	*P*-value
Admission status^a^	From home	101 (68.2)	40 (91.9)	1	
	Referred	47 (31.8)	36 (8.1)	5.27 (3.25–8.57)	0.000
Age group	≤19 years	9 (6.1)	29 (6.5)	1.1 (.50–2.38)	0.819
	20–34 years	125 (85.5)	368 (82.9)	1	
	≥35 years	14 (9.5)	47 (10.6)	1.14 (0.61–2.14)	0.819
Level of education^b^	Primary	3 (5.6)	7 (4.3)	0.69 (0.16–2.91)	0.613
	Secondary	30 (55.6)	85 (52.1)	0.84 (0.44–1.59)	0.589
	Tertiary	21 (38.9)	71 (43.6)	1	
Place of residence	Rural	33 (22.3)	89 (20.0)	1.15 (0.71–1.19)	0.557
	Urban	115 (77.7)	355 (80.0)	1	
Employment status	No	70 (47.3)	218 (49.1)	1.15 (0.71–1.19)	0.704
	Yes	78 (52.7)	226 (50.9)	1	
Occupation^c^	Employed	15 (10.8)	80 (19.0)	1.79 (0.93–3.47)	0.084
	Self employed	63 (45.3)	150 (19.0)	0.90 (0.58–1.47)	0.644
	Unemployed	61 (43.9)	190 (45.2)	1	

*CI* confidence interval, Odd ratio. ^a^Information on referral status was only available for 76 controls. ^b^Information on level of education was available for only 54 cases and 163 controls. ^c^Information on occupation was missing for 9 cases and 24 controls

### Medical risk factors of stillbirth

Preeclampsia (OR 4.99; 95% CI: 2.34–10.62, p = 0.000), maternal mild anaemia (OR 1.82; 95% CI: 0.13–2.59, *p* = 0.013) and moderate anaemia (OR 1.86; 95% CI: 0.11–3.10, *p* = 0.018) were found to be significantly associated with stillbirth. Chronic hypertension and maternal anaemia increased the odds of having a stillbirth but these associations were not statistically significant (Table 3).

**Table 3:** Medical factors associated with stillbirth (bivariate analysis)

Variables	Categories	Cases N (%)	Controls N (%)	OR (95% CI)	*P*-value
Anaemia^a^	No	43 (34.7)	209 (49.4)	1	0.473
	Mild	46 (46)	123 (29.1)	1.82 (0.13–2.59)	0.013
	Moderate	34 (27.4)	89 (21.0)	1.86 (0.11–3.10)	0.018
	Severe	1 (0.8)	2 (0.5)	2.43 (0.22–27.41)	0.473
Preeclampsia	No	130 (87.8)	432 (97.3)	1	
	Yes	18 (12.2)	12 (2.7)	4.99 (2.34–10.62)	0.000
Eclampsia	No	148 (100)	443 (99.8)	0.33 (0–0)	0.563
	Yes	0 (0.0)	1 (0.2)	1	
Chronic hypertension	No	147 (99.3)	443 (99.8)	1	
	Yes	1 (0.7)	1 (0.2)	3.01 (0.19–48.48)	0.436
Malaria	No	135 (91.2)	407 (91.7)	1	
	Yes	13 (8.8)	37 (8.3)	1.06 (0.55–2.05)	0.865
HIV	No	143 (96.6)	427 (96.2)	1	
	Yes	5 (3.4)	17 (3.8)	1.14 (0.32–2.42)	0.802
Alcohol consumption	No	127 (85.8)	383 (86.3)	1	
	Yes	21 (14.2)	61 (13.7)	1.04 (0.61–1.77)	0.891

*CI* confidence interval, *HIV* human immunodeficiency virus, *OR* odd ratio. ^a^Information on anaemia was missing for 24 cases and 21 controls

### Obstetrics and foetal risk factors of stillbirth

Not attending ANC (OR 17.75; 95% CI: 3.89–81.03, *p* = 0.000), attending ANC at a primary health care facility (OR 1.8; 95% CI: 1.22–2.64, *p* = 0.003), booking ANC visit after 12 weeks (OR 8.11; 95% CI: 1.94–33.85, *p* = 0.004), placenta abruption (OR 35.57; 95% CI: 4.55–277.98, *p* = 0.001), PROM (OR 2.62; 95% CI: 1.26–5.47, *p* = 0.010), oligohydramnios (OR 9.34; 95% CI: 1.56–46.78, *p* = 0.007), preterm birth (OR 6.17; 95% CI: 3.88–9.81, *p* = 0.000), LBW (OR 7.76; 95% CI: 4.65–12.96, p = 0.000) and twin pregnancy (OR 3.13; 95% CI: 1.95–8.5, *p* = 0.025) were associated with stillbirth in univariate analysis. Malpresentation was protective of stillbirth (OR 0.75; 95% CI: 0.16–3.56, p = 0.007) (Table 4).

**Table 4:** Obstetric factors associated with stillbirth (bivariate analysis)

Variable	Categories	Cases N (%)	Controls N (%)	OR (95% CI)	*P*-value
Parity	Grand multiparous (≥5)	4 (2.7)	8 (1.8)	0.660 (0.194–2.239)	0.505
	Multiparous (2–4)	47 (31.8)	142 (32.0)	0.99 (0.67–1.49)	0.988
	Primiparous (1)	97 (65.5)	294 (66.2)	1	
Booking status	Not booked	11 (7.4)	2 (0.5)	17.75 (3.89–81.03)	0.000
	Booked	137 (92.6)	442 (99.5)	1	
Place of ANC^a^	Primary care centre	74 (53.2)	169 (38.8)	1.8 (1.22–2.64)	0.003
	Secondary care centre	65 (46.8)	267 (61.2)	1	
GA at first ANC	> 12 weeks	135 (98.5)	389 (88.4)	8.11 (1.94–33.85)	0.004
Visit^b^	≤12 weeks	2 (1.5)	51 (11.6)	1	
History of stillbirth	No	146 (98.6)	439 (98.9)	1	
	Yes	2 (1.4)	5 (1.1)	1.21 (0.74–1.95)	0.448
Placenta praevia	No	144 (97.3)	439 (98.9)	1	
	Yes	4 (2.7)	5 (1.1)	2.439 (0.65–9.21)	0.188
Placenta abruption	No	137 (92.6)	443 (99.8)	1	
	Yes	11 (7.4)	1 (0.2)	35.57 (4.55–277.98)	0.001
PROM	No	134 (90.5)	427 (96.2)	1	
	Yes	14 (9.5)	17 (3.8)	2.62 (1.26–5.47)	0.010
GA at rupture of membranes	28–37 weeks	10 (71.4)	10 (2.3)	5.71 (0.92–35.47)	0.061
	37–42	4 (28.6)	9 (47.4)	1	
Chorioamnionitis	No	146 (98.6)	442 (99.5)	1	
	Yes	2 (1.4)	2 (0.5)	3.03 (0.42–21.68)	0.270
Oligohydramnios	No	142 (95.9)	442 (99.5)	1	
	Yes	6 (4.1)	2 (0.5)	9.34 (1.86–46.78)	0.007
Malpresentation	No	146 (98.6)	436 (98.2)	1	
	Yes	2 (1.4)	8 (1.8)	0.76 (0.16–3.56)	0.007
Dystocia	No	138 (93.2)	433 (97.5)	1	
	Yes	10 (6.8)	11 (2.5)	2.85 (1.19–6.86)	0.019
Acute foetal distress	No	144 (97.3)	434 (97.7)	1	
	Yes	4 (2.7)	10 (2.3)	1.21 (0.37–3.90)	0.755
Sex	Male	77 (52.0)	229 (51.6)	0.98 (0.67–1.43)	0.924
	Female	71 (56.1)	215 (48.4)	1	
Number of babies^c^	One	133 (94.3)	416 (98.1)	1	
	Twin	8 (5.7)	8 (1.9)	3.13 (1.15–8.5)	0.025
Birth weight (g)	< 2500	58 (39.2)	34 (7.7)	7.76 (4.65–12.96)	0.000
	2500–4000	83 (56.1)	364 (82.0)	1	
	> 4000	7 (4.7)	46 (10.4)	0.67 (0.65–3.44)	0.340
Gestational age (weeks)	28–37	68 (45.9)	55 (12.4)	6.17 (3.88–9.81)	0.000
	37–42	70 (47.3)	341 (76.8)	1	
	> 42	10 (6.8)	48 (10.8)	1.02 (0.48–2.04)	0.968

*ANC* antenatal care, *GA* gestational age, *PROM* premature rupture of membranes, *OR* odd ratio. ^a^Information on place of ANC was missing for 9 cases and 8 controls. ^b^Information on GA at start of ANC was missing for 11 cases and 8 controls. ^c^Information on number of babies was missing for 7 cases and 20 controls

### Factors independently associated with stillbirth after adjusting for confounders (multivariate analysis)

The factors that were independently associated with stillbirth were: referral from another health facility (AOR 3.95; 95% CI: 1.85–6.58, *p* = 0.000), late booking visit after 12 weeks (AOR 13.64; 95% CI: 1.49–124.83, *p* = 0.021), preeclampsia (AOR 3.21; 95% CI: 1.23–8.35, *p* = 0.01), placental abruption (AOR 21.46; 95% CI: 2.36–194.77, *p* = 0.006), moderate anaemia (AOR 2.04; 95% CI: 1.09–3.83, *p* = 0.03), labour dystocia (AOR 5.37; 95% CI: 1.77–15.92, *p* = 0.003), low birth weight (AOR 5.27; 95% CI: 2.62–10.62, *p* = 0.000), and preterm delivery (AOR 2.81; 95% CI: 1.48–3.35, *p* = 0.002). Term babies (AOR 0.37; 95% CI: 0.19–0.71, p = 0.003) and post-term babies (AOR 0.32; 95% CI: 0.12–0.91, *p* = 0.032) were less likely to be stillborn than preterm babies. Similarly, babies with normal weight (AOR 0.19; 95% CI: 0.1–0.38, p = 0.000) and macrosomic babies (AOR 0.17; 95% CI: 0.05–0.54. p = 0.003) were less likely to be stillborn than low birth weight babies (Table 5).

**Table 5:** Factors independently associated with stillbirth (multivariate analysis)

Variables	AOR	95% CI	*P*-value
Admission status			
Referred from another health facility	3.95	1.89–6.58	0.000
From home	1	.	.
Start of antenatal care visits			
> 12 weeks	13.64	1.49–124.83	0.0215
≤ 12 weeks	1	.	.
Place of antenatal care visits			
Secondary health care	0.1	0.43–1.13	0.141
Primary health care	1	.	.
Preeclampsia			
Yes	3.21	1.23–8.35	0.01
No	1	.	.
Placental abruption			
Yes	21.46	2.36–194.77	0.006
No	1	.	.
Anaemia			
Severe Anaemia	1.56	0.11–21.69	0.742
Moderate Anaemia	2.04	1.09–3.83	0.026
Mild Anaemia	1.64	0.92–2.91	0.092
No Anaemia	1		
PROM			
Yes	1.44	0.48–4.36	0.51
No	1	.	.
Oligohydramnios			
Yes	1.23	0.15–9.97	0.844
No	1	.	.
Mal presentation			
Yes	1		
No	2.06	0.34–1.44	0.43
Number of babies			
One	0.60	0.17–2.15	0.43
Two	1	.	.
Birth weight (g)			
< 2500	5.27	2.62–10.62	0.000
> 4000	0.81	0.24–2.70	0.734
2500–4000	1		
Labour dystocia			
Yes	5.31	1.77–15.92	0.003
No	1	.	.
Age of pregnancy (weeks)			
28–37	2.81	1.48–5.35	0.002
> 42	0.92	0.38–2.24	0.848
37–42	1		

*AOR* adjusted odd ratio, *g* gram, *PROM* premature rupture of membranes

## Discussion

This study presents stillbirth rates and risk factors in a semi-urban setting in Cameroon, which may represent the true picture of an LIC such as Cameroon. The global stillbirth rate in both hospitals was 35.03 per 1000 births. The stillbirth rates in both BRH and LRH were similar (33.72 and 36.45 per 1000 births respectively). They were both higher than 25.6 per 1000 births reported by the WHO for Cameroon in 2009 and 25.8 per 1000 births reported in a similar study in a tertiary health care centre in Douala, Cameroon in 2017, which used a lower cut-off 22 completed weeks or 500 g [11, 12]. Our study was carried out in secondary healthcare facilities and our results are consistent with the observations that stillbirth rates vary widely within regions and are more common in resource-limited settings [10–12]. However, a similar study in Nigeria that used the same cut-off of 28 weeks or 1000 g reported stillbirth rates of 46.9 per 1000 births [11]. This difference may be due to the difference in health care indices between regions. In our study, up to 51.4% of the stillbirths were fresh stillbirths, while 48.6% were macerated. Fresh stillbirth may be a surrogate for intrapartum stillbirth, most of which are preventable with good intrapartum care [13].

Being referred from another facility increased the possibility of stillbirth about four times. Most of these centres have little equipment for intrapartum monitoring and late referral could account for this observation. The findings of this study were consistent with results of a similar study carried out in Douala, Cameroon and India [10, 14].

The odds of stillbirth were over three times higher among women with preeclampsia. The association between preeclampsia and stillbirth was also demonstrated by a good number of similar studies in low-income countries [10, 11]. This study shows poor antenatal care uptake, particularly late booking. A study in the same hospitals reported a mean gestational age at booking visit of 19.2 weeks, with the majority of women presenting after the first trimester mainly due to financial constraints [15]. This makes it difficult to identify women with or at risk of preeclampsia early enough, which is often asymptomatic. This can lead to delay in identification and management of women at risk. A similar study in Douala Referral Hospital with a lower mean gestational age at booking visit of 14 weeks did not show any significant increase in odds of stillbirth among women with preeclampsia [10, 16].

Our study showed an increase in odds of stillbirth with increasing severity of maternal anaemia in pregnancy. Suleiman et al. also showed an increase in the odds of anaemia from mild to severe [11]. Our study, however, did not show any significant increase in odds of stillbirth among women with severe anaemia. This could be due to the differences in study design and health indices. Anaemia is associated with increased risks of intrauterine growth restriction (IUGR), and consequently intrauterine foetal death (IUFD) [17]. To prevent anaemia and its complications in pregnancy, a daily 30 mg to 60 mg of ferrous sulfate and 0.4 mg of folic acid supplementation are recommended by WHO [18].

Starting ANC after 12 weeks increased the odds of stillbirth over 13-fold. Few women (9.2%) started ANC before 12 weeks of gestation. The mean gestational age at booking visit among cases (21.60 ± 5.04) was greater than that for the control group (19.51 ± 5.8) (*p* = 0.000). Complications like preeclampsia can be identified early with early booking. Also, women who begin ANC early may have a higher tendency to attend more antenatal care visits. This will result overall in better care and will significantly reduce the risk of stillbirths [19].

The risk of stillbirth was 21 times higher among women who had placenta abruption. Similar studies by, Suleiman et al. (in Northern Nigeria) and Nazli et al. (in Pakistan) reported that this risk was 257 and 136 times respectively [11, 20]. These differences could be due to the differences in the severity and time of presentation that were not accounted for. However, antepartum haemorrhage (including placenta praevia and abruption) is a leading cause of perinatal and maternal mortality [21]. One important factor in the context of this study is the time needed to secure and critically screen blood for compatibility and infections like HIV, whose prevalence among adults in Cameroon is about 4.3%, particularly among women (5.6%) [22]. This may reduce the chance of salvaging mothers and babies during this life-threatening rapidly developing anaemia, accounting for the significant increase in the odds of stillbirth in placental abruption.

Stillbirths were over five times more likely to occur among women with difficult labour (dystocia). Labour lasting more than 12 h has been reported in Yaoundé, Cameroon to increase the risk of intrapartum stillbirth [9]. Intrapartum foetal deaths are less frequent in areas where women receive good quality care during childbirth [13]. The study health facilities have no cardiotocograph for continuous electronic monitoring of maternal and foetal wellbeing during labour. The facilities use the modified WHO partograph and the Pinard stethoscope or foetal Doppler for labour monitoring. This makes identifying complications that arise at any time during the dynamic process of labour difficult, hence delay in decision-making.

Preterm babies were about three times more likely to be stillborn than term babies. This is consistent with other similar studies [10, 12]. Children born preterm are premature and may not be well adapted to withstand labour and transition to extra-uterine life compared to children born at term.

The odds of stillbirth were over five times higher for low birth weight babies than babies with normal weight. Other studies show that a baby with low birth weight has a higher chance of being stillborn [8, 10, 11]. Low birth weight babies like preterm babies may not be well adapted for the transition to extra-uterine life. Also important is the associated adverse intrauterine environment before foetal death, probably caused by other risk factors. Low foetal weight for gestational age can, therefore, be an indication of on-going foetal compromise from other serious complications, which should prompt the physician to further re-evaluate or refer, where necessary.

After adjusting for confounders, the place of ANC, PROM, oligohydramnios and the number of babies did not increase the odds of stillbirth and malpresentation was no more found to be significantly protective of stillbirth.

### Limitations and strengths of the study

#### Limitations

A good number of files of stillbirth had incomplete data, thereby excluding them from the analysis. Furthermore, our study was hospital-based, and stillbirths that occurred at home were not included since these may not be reported. This could hide the true picture of the burden of stillbirth.

#### Strengths

This study was carried out in the main referral hospitals in the Southwest Region of Cameroon, which receive more pregnant women and deliveries than other health facilities in the region. Finally, the study was carried out in a semi-urban area, making the results representative of the picture in a low-income country like Cameroon.

## Conclusion

Stillbirth rates at the BRH and LRH are high compared to the targets set by WHO’s Every New-Born Action Plan in all countries by 2030 and current WHO estimates for Cameroon [12].

Risk factors associated with stillbirth include referral from another health facility, preeclampsia, maternal anaemia, factors related to antenatal care coverage and quality or management of labour and delivery (late booking visit, placental abruption, labour dystocia) as well as foetal complications (preterm birth and low birth weight). We recommend electronic data storage in hospitals in Cameroon.

## Data Availability

The data supporting the conclusions in this article are included in the article.

## Abbreviations

ANC: Antenatal care
AOR: Adjusted odds ratio
BRH: Buea Regional Hospital
HIC: High Income Country
LBW: Low Birth Weight
LIC: Low-Income country
LRH: Limbe Regional Hospital
MIC: Middle Income Country
SBR: Stillbirth rates
WHO: World Health Organisation
